# MALDI-TOF MS Using a Custom-Made Database, Biomarker Assignment, or Mathematical Classifiers Does Not Differentiate *Shigella* spp. and *Escherichia coli*

**DOI:** 10.3390/microorganisms10020435

**Published:** 2022-02-14

**Authors:** Maaike J. C. van den Beld, John W. A. Rossen, Noah Evers, Mirjam A. M. D. Kooistra-Smid, Frans A. G. Reubsaet

**Affiliations:** 1 Centre for Infectious Disease Control, National Institute for Public Health and the Environment (RIVM), Antonie van Leeuwenhoeklaan 9, 3721 MA Bilthoven, The Netherlands; noah.evers@polpharmabiologics.com (N.E.); frans.reubsaat@rivm.nl (F.A.G.R.); 2 Department of Medical Microbiology and Infection Prevention, University Medical Center Groningen, University of Groningen, Hanzeplein 1, 9713 GZ Groningen, The Netherlands; john.rossen@gmail.com (J.W.A.R.); m.kooistra@certe.nl (M.A.M.D.K.-S.); 3 Department of Pathology, University of Utah School of Medicine, 30 N 1900 E, Salt Lake City, UT 84132, USA; 4 Laboratory of Clinical Microbiology and Infectious Diseases, Isala Hospital, Dr. Van Heesweg 2, 8025 AB Zwolle, The Netherlands; 5 Department of Medical Microbiology, Certe, Van Swietenlaan 2, 9728 NZ Groningen, The Netherlands

**Keywords:** MALDI-TOF MS, *Shigella* spp., *Escherichia coli*, EIEC, custom-made database, biomarker assignment, machine-learning classifiers

## Abstract

*Shigella* spp. and *E. coli* are closely related and cannot be distinguished using matrix-assisted laser desorption-ionization time-of-flight mass spectrometry (MALDI-TOF MS) with commercially available databases. Here, three alternative approaches using MALDI-TOF MS to identify and distinguish *Shigella* spp., *E. coli*, and its pathotype EIEC were explored and evaluated using spectra of 456 *Shigella* spp., 42 *E. coli*, and 61 EIEC isolates. Identification with a custom-made database resulted in >94% *Shigella* identified at the genus level and >91% *S. sonnei* and *S. flexneri* at the species level, but the distinction of *S. dysenteriae*, *S. boydii*, and *E. coli* was poor. With biomarker assignment, 98% *S. sonnei* isolates were correctly identified, although specificity was low. Discriminating markers for *S. dysenteriae*, *S. boydii*, and *E. coli* were not assigned at all. Classification models using machine learning correctly identified *Shigella* in 96% of isolates, but most *E. coli* isolates were also assigned to *Shigella*. None of the proposed alternative approaches were suitable for clinical diagnostics for identifying *Shigella* spp., *E. coli*, and EIEC, reflecting their relatedness and taxonomical classification. We suggest the use of MALDI-TOF MS for the identification of the *Shigella* spp./*E. coli* complex, but other tests should be used for distinction.

## 1. Introduction

The *E. coli* pathotype entero-invasive *E. coli* (EIEC) is thought to cause the same disease as *Shigella* spp. [1]. This pathotype consists of isolates that possess some of the *E. coli* phenotypic characteristics and the invasive nature of *Shigella* spp. [2,3]. EIEC harbors the same virulence markers as *Shigella* spp. that are used in molecular diagnostics to detect both *Shigella* spp. and EIEC but are not suitable to distinguish them [4]. *Shigella* spp. and *E. coli* are described to belong to one taxonomic species genetically, but classification in two genera is maintained for practical and taxonomic reasons [2,3,4,5]. Therefore, differentiation is challenging and is historically performed using phenotypical tests, serotyping, and the determination of virulence markers using PCR [6,7]. Multiple researchers have designed molecular methods to distinguish *Shigella* and *E. coli*, and EIEC in particular [8,9,10]. Although most molecular methods are ≥95% accurate using the initially selected set of isolates, they appeared not to be reliable when these methods were used for additional isolates [3,11].

Most clinical laboratories currently use matrix-assisted laser-desorption ionization time-of-flight mass spectrometry (MALDI-TOF MS) to identify bacteria in a routine diagnostic setting. Commercially available databases, such as MALDI biotyper^®^ in combination with the MALDI Security-Relevant (SR) library^®^ (Bruker Daltonik GmbH, Bremen, Germany) and VITEK^®^ MS (BioMérieux, Marcy-l’Etoile, France) can distinguish *Shigella* spp. and *E. coli* from other *Enterobacteriaceae.* However, they cannot distinguish between the different *Shigella* species and *E. coli*, including the EIEC pathotype [12].

The development of custom-made databases to identify bacteria using MALDI-TOF MS as an alternative to commercially available databases proved successful for multiple species before [13,14,15]. Most notably, an earlier study developed a custom-made database to identify and distinguish *Shigella* spp. and *E. coli* specifically. However, EIEC isolates were not included in their database [16]. Using a database approach, comparisons of unknown isolates to spectra in a database comprise the whole spectra for species identification. However, for closer related groups, a more subtle approach can be essential, in which variations within the spectra are examined in the presence or absence of specific peaks as biomarkers [17,18]. The biomarker approach was used to type *E. coli* isolates before, with varying success rates [18]. These approaches mainly targeted a selection of isolates representing the pathotype entero- hemorrhagic *E. coli* (EHEC) or the highly virulent ST131 clone [18], although two studies used biomarker typing specifically for *Shigella* spp. and *E. coli*, without EIEC isolates [19,20]. One of those studies identified biomarkers outside the mass range of 2000–20,000 Da used in routine applications [20], and the other did not specify in which species the biomarkers were present or absent [19]. Besides determining the presence or absence of single biomarkers, patterns of these biomarkers can be investigated and recognized with machine-learning algorithms [21]. These machine-learning-based methods can establish classifiers for identifying groups within species of bacteria [22,23]. Moreover, these classifiers were developed to identify *Shigella* spp. and *E. coli* before, although EIEC isolates were not included [19].

In this study, the ability of MALDI-TOF MS was assessed for the distinction of the four *Shigella* species, EIEC, and non-invasive *E. coli* using alternatives for the commercially available databases. First, a custom-made database, including all *Shigella* species, *E. coli*, and EIEC isolates, was developed and evaluated. Second, biomarkers were assigned and evaluated, and third, classifier models based on machine learning were defined, applied, and evaluated.

## 2. Materials and Methods

### 2.1. Bacterial Isolates

A total of 559 isolates consisting of 36 *S. dysenteriae*, 156 *S. flexneri*, 32 *S. boydii*, 232 *S. sonnei*, 61 EIEC, and 42 other *E. coli* of human and animal origin comprising phylogroups A, B1, B2, and D [24] were used (Table 1). 

All isolates, except the references, were identified using a previously described culture-based identification algorithm [25]. They were divided into a set of training isolates (*n* = 288) and test isolates (*n* = 271), both having similar species and serotype distributions. The training set was used to construct the custom-made database, assign biomarkers, and define and train machine-learning classifier models. The test set was used to test all of these algorithms in duplicate, with both direct smear and ethanol-formic acid extraction application methods.

### 2.2. MALDI-TOF MS Preparation of Isolates

All isolates were grown overnight on Columbia Sheep Agar (CSA, Biotrading, Mijdrecht, The Netherlands) at 37 °C and were subsequently subjected to the direct smear method and the ethanol-formic acid extraction with silica beads as previously described [26]. Colonies or 1 µL extract were applied onto a polished steel plate, air-dried, and overlaid with 1 µL α-Cyano-4-hydroxycinnamic acid in 50% acetonitrile-2.5% trifluoroacetic acid (HCCA matrix). The samples were analyzed using a Bruker Microflex LT (Bruker Daltonik GmbH, Bremen, Germany) in a linear and positive mode, with 30–40% laser power and within a mass range of 2000–20,000 Da.

### 2.3. Database Development

**Figure 1:**
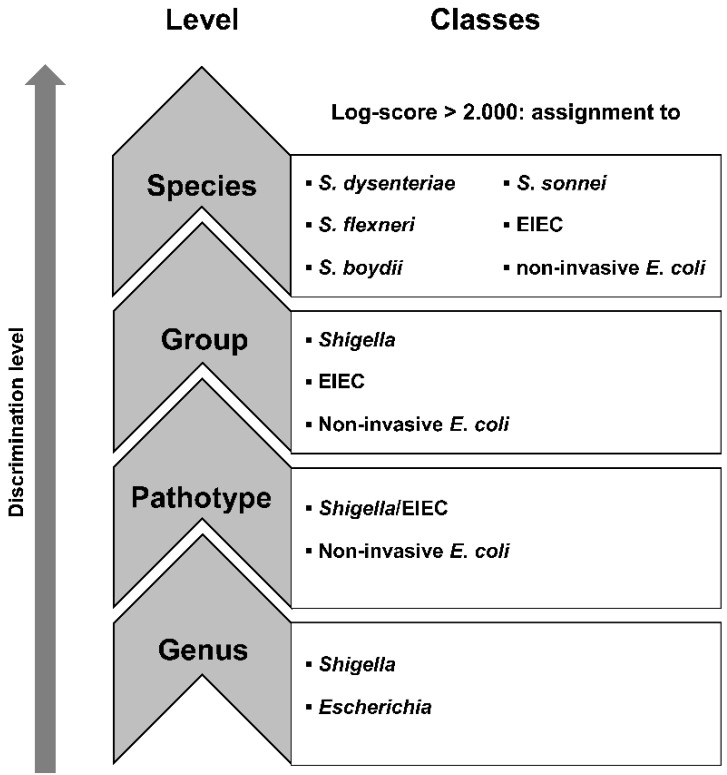
The classes in the different discrimination levels to which isolates were assigned.

The MSPs produced from 288 isolates in the training set were used to build a custom-made database with Maldi Biotyper OC V3.1.66 (Bruker Daltonik). In addition, a dendrogram to assess the relatedness of these MSPs was inferred using default settings. The isolates in the test set were identified using this custom-made database. Additionally, the test isolates were also identified using the commercially available Bruker MALDI Biotyper database (V8.0.0.0) and the Bruker Security-Relevant Library (V1.0.0.0) and using a combination of the commercial and custom-made databases. Quality of the results was indicated by a log-score, calculated by Maldi Biotyper 3.0 RTC: a log-score of 2.000–2.300 corresponds to “secure genus identification, probable species identification”, and a log-score of >2.300 corresponds to a “highly probable species identification”. Both duplicate spots were analyzed, the highest log-score of at least 2.000 was considered as the definitive MALDI-TOF MS identification, as is done in a routine workflow. If an isolate had a log-score < 2.000 caused by a poor spectrum, it was disregarded from further analysis. Isolates were then assigned to different discrimination levels “genus”, “pathotype”, “group”, and “species”, as displayed in Figure 1.

For accurate identification, only matches with database MSPs from the same species within a log-score range of 2.000–2.300 or >2.300 should be expected in one spot. Therefore, the ten MSPs from the database that produced the highest scores within a log-score of 2.000–2.300 or >2.300 per spot were determined. For each species identified with the culture-dependent identification algorithm, the median number of species resulting from MALDI-TOF MS and their quartile ranges per spot with a log-score of 2.000–2.300, or >2.300 were calculated and visualized using SPSS 24.0.0.1 (IBM, New York, NY, USA).

### 2.4. Biomarker Assignment and Principal Component Analysis

Spectra files from MSPs of 288 isolates in the training set were exported as mzXML files using Compassxport CXP3.0.5. (Bruker Daltonik) or exported via a batch process in Flexanalysis (Bruker Daltonik). A new database was created in Bionumerics v7.6.3 (Applied Maths NV, Sint-Martens-Laten, Belgium ) according to the manufacturers’ instructions. All raw spectra were imported into the Bionumerics database with x-axis trimming to a minimum of 2000 *m*/*z*. Baseline subtraction, noise computing, smoothing, baseline detection, and peak detection were performed with default settings. Spectra summarizing, peak matching, and peak assignment were performed according to instructions from Bionumerics [23].

In short, all raw spectra were summarized into isolate spectra. Peak matching was performed on isolate spectra using a constant tolerance of 1.9, a linear tolerance of 550, and a peak detection rate of 10%. Binary peak matching tables were exported to summarize the presence of peak classes on all discrimination levels, as depicted in Figure 1. Decision diagrams were produced for the levels genus, pathotype, and groups (Supplementary Figure S1a–c). The spectra files of isolates from the test set were imported and preprocessed in Bionumerics, using the same methods and settings as for the spectra from the isolates in the training set. Peak matching of test isolates was performed using the option “existing peak classes only” to compare the presence of peaks in the test isolates with peaks in the isolates from the training set. Decision diagrams (Supplementary Figure S1) and the presence or absence of peak masses as depicted in Table 2 were applied to assign unknown isolates from the test set according to the different levels, as shown in Figure 1.

By assigning biomarkers, only the presence and absence of peaks were investigated. To assess quantitative peak data such as peak intensity and peak area, a principal component analysis (PCA) was performed on all isolates in the training set to visualize the position of isolates in three dimensions.

### 2.5. Presence of Biomarkers Identified in Previous Studies

All isolates in the training and test sets were examined for the unique masses (±500 ppm) found in the biomarker assignment to *Shigella* spp. and *E. coli* in previous studies [19,20]. Additionally, because peaks in our study were assigned at *m*/*z* values instead of masses only and because masses could be potentially charged with two electrons, this is corrected by examining the previously published masses divided by two (±500 ppm) [19,20].

### 2.6. Classifier Models Based on Machine Learning

Peak data of the summarized isolate spectra of the 288 isolates in the training set were used to define and train machine learning-based classifiers using Bionumerics v7.6.3 according to the manufacturers’ instructions. In short, peak matching with a constant tolerance of 1.9 and a linear tolerance of 550 was performed on isolate spectra on the different levels: genus, pathotype, group, and species. Classifiers were created at all levels using character values. Support vector machine (linear) learning was used as a scoring method in which *p*-values were used for ranking. The classifiers were trained and cross-validated to check their performance for identification. Subsequently, the classifier models were used to classify the unknown isolates in the test set at the different discrimination levels to evaluate their performance.

## 3. Results

### 3.1. Database Development

**Figure 2:**
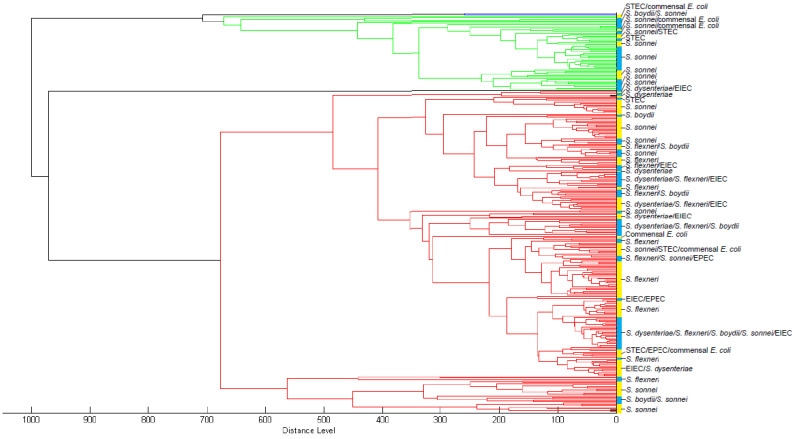
Dendrogram of MSPs of training isolates. Blue = cluster 1; green = cluster 2; red = cluster 3. Yellow/blue vertical band = manual cluster distinction at distance level 50–100 relative units with species designation using the culture-based identification algorithm.

All MSPs of 288 training isolates were added to a custom-made database. The relatedness of these MSPs is shown in a dendrogram (Figure 2). The Maldi Biotyper OC software recognized three large MSPs clusters that are not species-specific within this custom database. This did not change if clusters were assigned manually with a lower distance level at 50–100 relative units, indicating that similarity in spectrum profiles is distributed over the species level (Figure 2).

Additionally, the duplicate spots of test isolates using either the direct smear or extraction method resulted in a different species designation in 10–15% of the samples. 

**Figure 3:**
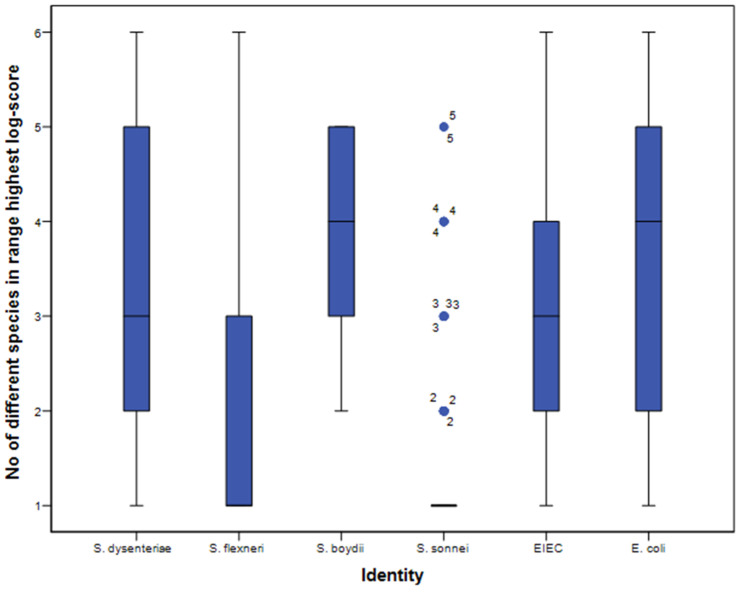
Number of different species in the first 10 matches per spot with the direct smear method. Identity (x-axis) was assigned using the culture-based identification algorithm. Black horizontal bars represent the median number of species; the 25–75% interquartile ranges are indicated by the blue vertical bars, and 5–95% intervals by the black vertical lines. Outliers are indicated with blue dots.

Furthermore, with an accurate distinction of species, one would not expect assignment to multiple species above the threshold of log-score 2.000. However, with both application methods, most isolates were assigned to several species with a log-score of 2.000–2.300 or >2.300 per spot, indicating no specificity at all (Figure 3).

One isolate from the test set (*S. boydii* serotype 13) showed a low-quality spectrum (log score 1.574–1.930), and one isolate (*S. dysenteriae* serotype 1) had initially been incorrectly stored, as this isolate was identified as *Corynebacterium diphtheriae* using the Bruker databases. Both these isolates were ignored in further analyses. All other isolates had log-scores higher than 2.000, and percentages of MALDI-TOF MS identification concordant with the original identification on all discrimination levels were as displayed in Table 3. With the Bruker databases only, percentages of correctly identified *Shigella* spp. on all discrimination levels are low, ranging from 6% to 45% correct designations, both for the direct smear and extraction methods (Table 3). In contrast, 90–100% of *E. coli* isolates were correctly identified. When identification was based on the custom-made database with or without the Bruker databases, the percentage of correctly identified *E. coli* isolates decreased to a range of 29–71%. In contrast, *Shigella* spp. were correctly identified, ranging from 94% to 99% of cases on the genus, pathotype and group levels. In addition, 91–97% of *S. flexneri* and *S. sonnei* were correctly identified at the species level, in contrast to *S. dysenteriae* and *S. boydii*, for which the percentages of correct identification were low (Table 3).

### 3.2. Biomarker Assignment and Principal Component Analysis

The decision diagrams based on biomarkers assigned to the isolates in the training set were used to identify unknown isolates in the test set. Distinctive peaks on the species levels are summarized in Table 2. High percentages for correct identification of *S. sonnei* isolates were achieved at the species level using both the direct smear and the extraction method. However, the biomarkers are not specific for *S. sonnei*, as other species contain them as well. For other species, the identified biomarkers correctly identified isolates below 38%. Specific biomarkers were not detected for all the classes at the different discrimination levels, as depicted in Figure 1. Consequently, it was not possible to identify *S. dysenteriae*, *S. boydii*, and *E. coli* isolates at all because of the absence of discriminating peaks for these species (Table 3).

**Figure 4:**
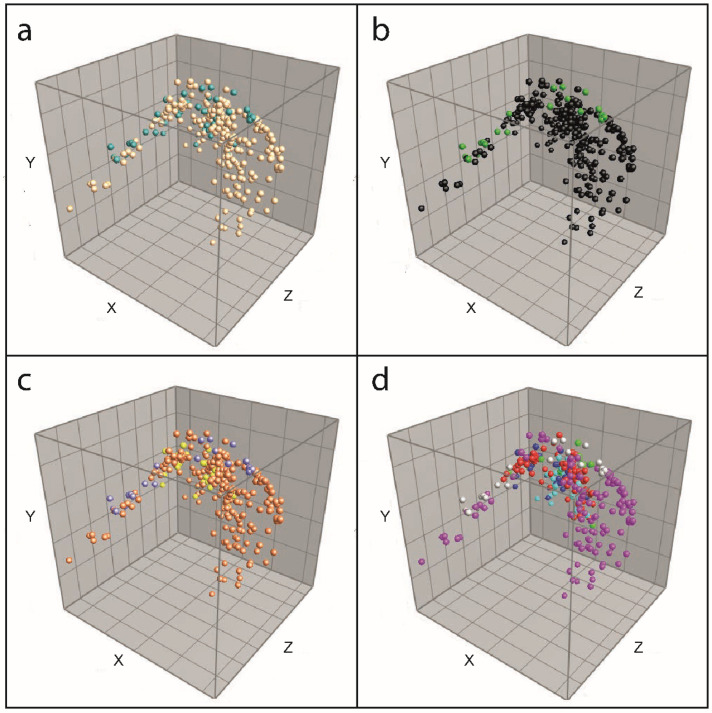
PCA of isolates in the training set. (**a**) Colored at genus level: beige = *Shigella*, teal = *Escherichia*; (**b**) Colored at pathotype level: black = *Shigella*/EIEC, green = *E. coli* (other than EIEC); (**c**) Colored at group level: orange = *Shigella* spp., yellow = EIEC, purple = *E. coli* (other than EIEC); (**d**) Colored at species level: light blue = *S. dysenteriae*, red = *S. flexneri*, green = *S. boydii*, pink = *S. sonnei*, blue = EIEC, light grey = Other *E. coli*.

In the PCA of the detected peaks in the isolates of the training set, one large cluster was formed, with a few outliers at both ends (Figure 4). If the isolates were colored according to their identity based on the culture-based identification method, separate groups of isolates were seen in none of the discrimination levels (Figure 4a–d).

### 3.3. Presence of Biomarkers Identified in Previous Studies

The specific biomarkers for *S. flexneri*, *S. sonnei*, and *E. coli* assigned by Everley et al. [20] were not present in any of the 559 isolates in this study when using an error limit of ±500 ppm. They were also not present if they were corrected for a charge with 2 electrons. A few biomarkers for *Shigella* spp. and *E. coli* described by Khot and Fisher [19] were present within a range of 500 ppm in isolates used in this study, i.e., 4163 Da, 7157 Da, 8326 Da, and 9227 Da, and corrected for a charge of 2 electrons, 5096 Da and 5752 Da.

### 3.4. Classifier Models Based on Machine Learning

Using the internal cross-validation of the classifiers at all discrimination levels, all but one class offered an accuracy of more than 87.5%. The only class with a lower accuracy (77%) was “Escherichia” at the genus discrimination level.

When using machine learning-based classifiers for identification, 96% of *Shigella* spp. isolates and 21% of the *E. coli* isolates from the test set were correctly identified at the genus level, using the direct smear application method and, respectively, 100% and 8% using the ethanol-formic acid extraction method (Table 3). Correct identification percentages for the pathotype, group, and species level were displayed in Table 3. Although more than 80% of *S. sonnei* isolates were correctly identified with the species classifier, specificity was low, as more than 70% of *S. flexneri* isolates were also identified as *S. sonnei*.

## 4. Discussion

Current commercially available MALDI-TOF MS databases cannot distinguish between *Shigella* spp. and *E. coli*. Therefore, three different alternatives were explored in this study. A custom-made database was developed, biomarkers were identified, and machine learning classification models were designed.

Compared to a previous study, our custom-made database assigned fewer *E. coli* isolates correctly [16]. This indicates that the inclusion of EIEC isolates in the custom-made database and the test set complicates the identification. Half of the EIEC isolates were assigned to one of the *Shigella* species, thereby decreasing the percentage of correctly identified *E. coli*. The poor performance of identifying *E. coli* with our custom-made database can result from an overrepresentation of *S. flexneri* and *S. sonnei*. A second custom-made database was developed to investigate this hypothesis, based on 17 isolates of each species, representing the diversity in serotypes. This database did not perform better or worse than the custom-made database that contained 288 MSPs (Supplementary Table S1), indicating that a more even distribution of species in the database does not improve the identification of *E. coli*. Although percentages of correct species assignments to *S. flexneri* and *S. sonnei* were high, other species were falsely assigned to them in our study and a previous study [16]. In the latter study, correct species identification was based on the majority rule that three out of four spots should indicate the same species. Besides the fact that the interpretation of four spots per isolate is not feasible in clinical diagnostics, this indicates that the assignment of species is based on probabilities rather than actual variations in spectra. Our study confirms this phenomenon because multiple species identifications within the same log-score range were made per spot. Moreover, 10–15% of duplicate spots resulted in different species assignments using commercially available and custom-made databases. Additionally, in the dendrogram inferred from the MSPs of the training set only into the custom-made database, the same species were not clustering together, indicating that the resulting database would not be capable of identifying the isolates from the test set correctly.

Another alternative approach for using commercially available databases is the detection of discriminating biomarkers. However, in our study, many isolates resulted in inconclusive identification, as specific biomarkers were not detected for most classes. Although more than 90% of *S. sonnei* isolates were identified at the species level, other species, such as *S. boydii* and *E. coli*, are also frequently falsely identified as *S. sonnei*. Moreover, when also analyzing peak intensity and area rather than just peak presence, the PCA showed that *Shigella* spp. and *E. coli* did not represent separated groups based on their biomarkers. In contrast, one large cluster with a few outliers was formed, demonstrating their genetic similarity. Furthermore, the absence of 85% of the masses assigned as biomarkers in a former study [15] in our isolates indicates that the detected biomarkers vary amongst isolate sets tested and that a stable variation per species is not observed. Consequently, we anticipate that the assignment of biomarkers based on yet another set of isolates will lead to even more diversity in biomarkers, demonstrating their unsuitability for distinct identification of *Shigella* spp., *E. coli*, and EIEC. In fact, peaks described in specific sets of isolates should not be considered as biomarkers if they are not detectable in (almost) all isolates of a species.

The use of classifier models based on machine learning resulted in comparable percentages of correctly identified *Shigella* on the genus level, i.e., ≥94%, as reported in other studies [19]. In our classifier model designed on the pathotype level, EIEC isolates were not incorporated in the class *E. coli*; correct identification was 67%, comparable to a previous study [19]. Nonetheless, the other remaining *E. coli* isolates were falsely classified as *Shigella*, both with our classifiers and with previously published ones [19], decreasing the specificity for identifying *Shigella*. Classifiers performed even less at the group and species level, and most species could not be identified at all. The poor performance of the classifier models may be caused by an overrepresentation of *S. flexneri* and *S. sonnei*, as discussed for the custom-made database in our study. Therefore, 17 isolates of each species were selected again, and alternative classifiers were designed. These classifiers did not perform better or worse than the classifiers designed using all 288 isolates in the training set, indicating that an absence of an even distribution of species was not the cause for poor identification with classifiers (Supplementary Table S1).

We used a substantially more extensive set of isolates than previous studies and included the *E. coli* pathotype EIEC. Another strength of our study was that multiple alternative approaches for identifying *Shigella* spp. and *E. coli* using MALDI-TOF MS were explored. Although *S. sonnei* and *S. flexneri* isolates were overrepresented in both the training and test sets, this distribution represents high-resource settings.

In conclusion, none of our explored alternative approaches for identifying *Shigella* spp., *E. coli*, and EIEC with MALDI-TOF MS were suitable to use in clinical diagnostics, as all rendered a poor distinction based on spectra or biomarkers. This poor discrimination merely reflects the problematic taxonomical classification of *Shigella* spp. and *E. coli* into two different genera and does not reflect MALDI-TOF MS’s performance as an identification technique in general. Therefore, we propose an identification algorithm in which MALDI-TOF MS is used to identify and differentiate *Shigella/E. coli* as a group from other *Enterobacteriaceae*, followed by tests other than MALDI-TOF MS to distinguish between the different *Shigella* species, *E. coli*, and specific *E. coli* pathotypes, including EIEC.

## Figures and Tables

**Table 1:** Isolates used in this study are divided into training and test sets.

Species and Serotype/O-Type	Training Set		Test Set	
	*n*	Origin	*n*	Origin
*S. dysenteriae* serotype 1	2	CIP 57.28^T^; A1	1	1 ci ^1^
*S. dysenteriae* serotype 2	5	A2, 4 ci ^1^	4	4 ci ^1^
*S. dysenteriae* serotype 3	5	AMC-43-G-93; 4 ci ^1^	3	3 ci ^1^
*S. dysenteriae* serotype 4	2	AMC 43-G-86; 1 ci ^1^	0	
*S. dysenteriae* serotype 5	1	AMC 43-G-84	0	
*S. dysenteriae* serotype 6	1	AMC 43-G-81	1	1 ci ^1^
*S. dysenteriae* serotype 7	1	AMC 43-G-76	1	1 ci ^1^
*S. dysenteriae* serotype 9	2	A58: 1646; 1 ci ^1^	1	1 ci ^1^
*S. dysenteriae* serotype 10	1	A2050-52	0	
*S. dysenteriae* serotype 12	2	2 ci ^1^	1	1 ci ^1^
*S. dysenteriae* serotype 14	1	NCTC 11867	0	
*S. dysenteriae* serotype 15	1	NCTC 11868	0	
Total number of *S. dysenteriae*	24		12	
*S. flexneri* serotype 1a	3	B1A; 2 ci ^1^	0	
*S. flexneri* serotype 1b	5	B1B; 4 ci ^1^	5	5 ci ^1^
*S. flexneri* serotype 1c	4	4 ci ^1^	3	3 ci ^1^
*S. flexneri* serotype 2a	32	CIP 82.48^T^; B2A; 30 ci ^1^	32	32 ci ^1^
*S. flexneri* serotype 2b	1	B2B	3	3 ci ^1^
*S. flexneri* serotype 3a	2	B3A; 1 ci ^1^	14	14 ci ^1^
*S. flexneri* serotype 3b	2	B3B; B3C	3	3 ci ^1^
*S. flexneri* serotype 4a	1	B4A	4	4 ci ^1^
*S. flexneri* serotype 4av	4	5 ci ^1^	0	
*S. flexneri* serotype 4b	1	B4B	0	
*S. flexneri* serotype 4c	3	3 ci ^1^	0	
*S. flexneri* serotype 5b	1	B5	1	1 ci ^1^
*S. flexneri* serotype 6	10	B6; 9 ci ^1^	10	10 ci ^1^
*S. flexneri* serotype X	1			
*S. flexneri* serotype Y	2	2 ci ^1^	2	2 ci ^1^
*S. flexneri* serotype Yv	2	2 ci ^1^	0	
*S. flexneri* provisional	5	5 ci ^1^	0	
Total number of *S. flexneri*	79		77	
*S. boydii* serotype 1	2	AMC-43-G-58; 1 ci ^1^	3	3 ci ^1^
*S. boydii* serotype 2	3	CIP 82.50^T^; P288; 1 ci ^1^	4	4 ci ^1^
*S. boydii* serotype 3	1	D1	0	
*S. boydii* serotype 4	2	AMC-43-G-63; 1 ci ^1^	2	2 ci ^1^
*S. boydii* serotype 5	2	P143; 1 ci ^1^	0	
*S. boydii* serotype 6	1	CDC 9771 (D19)	0	
*S. boydii* serotype 7	1	AMC 4006 (Lavington)	0	
*S. boydii* serotype 8	0		1	1 ci ^1^
*S. boydii* serotype 9	1	1296/7	0	
*S. boydii* serotype 10	1	430	1	1 ci ^1^
*S. boydii* serotype 11	1	34	0	
*S. boydii* serotype 12	0		1	1 ci ^1^
*S. boydii* serotype 13	0		1	1 ci ^1^
*S. boydii* serotype 14	0		1	1 ci ^1^
*S. boydii* serotype 15	1	CDC C-703	0	
*S. boydii* serotype 18	1	1 ci ^1^	1	1 ci ^1^
Total number of *S. boydii*	17		15	
*S. sonnei*	117	CIP 82.49^T^; 116 ci ^1^	115	115 ci ^1^
EIEC	30	DSM 9027; DSM 9028; CCUG 11335; CCUG 38080; CCUG 38092; CCUG 38093; EW227; 1624-56; 1184-68; 145/46; L119B-10; 19 ci ^1^	31	31 ci ^1^
Other *E. coli* pathotypes (human)	11	7 STEC ci ^1^, 4 EPEC ci ^1^	11	8 STEC ci ^1^; 3 EPEC ci ^1^
Other *E. coli* pathotypes (animal) ^2^	10	5 mussel, 3 pigeon, 2 turkey	10	4 mussel, 3 pigeon, 2 turkey, 1 oyster

^1^ ci = clinical isolate. ^2^ isolated from animals, all other numbers are reference isolates.

**Table 2:** Discrimination scheme of biomarkers, percentage of isolates in the training set with specific biomarkers.

Biomarkers (*m*/*z*)		2691	2877	3129	3636	3647	3930	3939	4163	4189	4368	4501	4769	4775
*S. dysenteriae*	(*n* = 24)	92	4	100	0	0	0	100	0	100	100	100	0	100
*S. flexneri*	(*n* = 46)	100	0	100	0	63	53	18	1	94	97	99	22	97
*S. boydii*	(*n* = 17)	88	0	100	18	0	0	94	0	100	88	100	18	100
*S. sonnei*	(*n* = 117)	56	49	59	22	0	1	56	17	89	68	56	23	98
EIEC	(*n* = 31)	100	0	100	3	6	0	97	0	97	100	94	26	97
Other *E. coli*	(*n* = 21)	52	24	52	71	0	5	62	38	90	67	57	67	100
**Biomarkers (*m*/*z*)**	**4784**	**5156**	**5239**	**5386**	**5415**	**6262**	**6322**	**6412**	**6488**	**7275**	**7295**	**7715**	**7868**	**7879**
*S. dysenteriae*	100	100	8	92	52	100	100	0	0	0	0	0	0	100
*S. flexneri*	76	97	55	99	45	99	99	4	42	1	83	0	41	23
*S. boydii*	76	94	18	88	59	100	88	18	6	18	0	6	12	88
*S. sonnei*	69	86	27	73	0	74	62	13	1	17	0	28	18	75
EIEC	71	100	39	100	42	94	97	19	0	23	6	3	16	84
Other *E. coli*	19	86	0	67	0	57	67	48	10	52	0	24	43	33
**Biomarkers (*m*/*z*)**	**8326**	**8370**	**8379**	**9002**	**9227**	**9535**	**9546**	**9563**	**9739**	**10,300**	**10,310**	**10,488**	**10,934**	
*S. dysenteriae*	0	0	100	100	0	0	100	100	0	0	100	0	0	
*S. flexneri*	5	4	90	94	4	17	82	92	8	9	86	0	0	
*S. boydii*	0	18	82	88	12	18	82	88	6	18	82	0	12	
*S. sonnei*	15	15	82	38	12	16	85	54	15	13	70	34	36	
EIEC	6	16	77	87	13	23	77	81	6	19	77	0	0	
Other *E. coli*	43	38	38	24	48	48	62	38	43	48	48	0	5	
	**8326**	**8370**	**8379**	**9002**	**9227**	**9535**	**9546**	**9563**	**9739**	**10,300**	**10,310**	**10,488**	**10,934**	

**Table 3:** Correct identification results of isolates from the test set.

Correct Identification with MALDI-TOF, Direct Smear											Correct Identification with MALDI-TOF, Ethanol									
	Bruker Databases ^1^, *n* (%)		Custom Databases ^1^, *n* (%)		Bruker Databases ^1^ + Custom, *n* (%)		Biomarker Assignment, *n* (%)		Classifier Models, *n* (%)		Bruker Databases ^1^, *n* (%)		Custom Databases ^1^, *n* (%)		Bruker Databases ^1^ + Custom, *n* (%)		Biomarker Assignment, *n* (%)		Classifier Models, *n* (%)	
**Genus**																				
*Shigella* (*n* = 217)	19	(9)	205	(94)	205	(94)	10	(5)	209	(96)	12	(6)	207	(95)	205	(94)	15	(7)	217	(100)
*E. coli* (*n* = 52)	49	(94)	26	(50)	29	(56)	NA		11	(21)	47	(90)	35	(67)	37	(71)	NA		4	(8)
Unassigned ^2^	2	(1)	1	(0.4)	3	(1)	257	(96)	0	(0)	1	(0.4)	0	(0)	3	(1)	250	(93)	0	(0)
**Pathotype**																				
*Shigella*/EIEC (*n* = 248)	NA		233	(94)	241	(97)	217	(88)	145	(58)	NA		245	(99)	242	(98)	225	(92)	147	(59)
Other *E. coli* (*n* = 21)	21	(100)	6	(29)	10	(48)	NA		14	(67)	21	(100)	11	(52)	13	(62)	NA		6	(29)
Unassigned ^2^	2		1	(0.4)	3	(1)	46	(17)	0	(0)	0	(0)	0	(0)	3	(1)	27	(10)	0	(0)
**Group**																				
*Shigella* (*n* = 217)	19	(9)	205	(94)	205	(94)	193	(89)	131	(60)	12	(6)	207	(95)	205	(94)	195	(90)	134	(62)
EIEC (*n* = 31)	NA		9	(29)	8	(26)	NA		2	(6)	NA		19	(61)	19	(61)	NA		0	(0)
Other *E. coli* (*n* = 21)	21	(100)	6	(29)	10	(48)	NA		13	(62)	21	(100)	11	(52)	13	(62)	NA		7	(33)
Unassigned ^2^	2	(1)	1	(0.4)	3	(1)	49	(23)	0	(0)	1	(0.4)	0	(0)	3	(1)	36	(13)	0	(0)
**Species**																				
*S. dysenteriae* (*n* = 11)	5	(45)	5	(45)	5	(45)	0	(0)	0	(0)	4	(36)	7	(64)	6	(55)	0	(0)	0	(0)
*S. flexneri* (*n* = 77)	NA		70	(91)	70	(91)	24	(31)	6	(8)	NA		73	(95)	73	(95)	30	(39)	3	(4)
*S. boydii* (*n* = 14)	NA		1	(7)	0	(0)	0	(0)	0	(0)	NA		0	(0)	0	(0)	0	(0)	0	(0)
*S. sonnei* (*n* = 115)	NA		110	(96)	110	(96)	113	(98)	92	(80)	NA		112	(97)	112	(97)	108	(94)	101	(88)
EIEC (*n* = 31)	NA		9	(29)	8	(26)	0	(0)	1	(3)	NA		19	(61)	19	(61)	1	(3)	3	(10)
Other *E. coli* (*n* = 21)	21	(100)	6	(29)	10	(48)	0	(0)	12	(57)	21	(100)	11	(52)	13	(62)	0	(0)	4	(19)
Unassigned ^2^	2	(1)	1	(0.4)	3	(1)	85	(32)	0	(0)	1	(0.4)	0	(0)	3	(1)	97	(36)	0	(0)

NA = not applicable, as no discriminating peaks were assigned to these classes. ^1^ Bruker MALDI Biotyper database (V8.0.0.0) and the Bruker Security-Relevant Library (V1.0.0.0). ^2^ Number of isolates that could not be assigned to a class.

## Data Availability

The data presented in this study are available on request from the corresponding author.

## Supplementary Materials

The following supporting information can be downloaded at: https://www.mdpi.com/article/10.3390/microorganisms10020435/s1, Supplementary Figure S1: Decision diagrams of assigned biomarkers; Supplementary Table S1: Comparison of performance of a custom-made database and classifiers based on all 288 isolates or based on an even distribution of 17 isolates for each species.
