# Supplementary material for: MALDI-TOF MS Using a Custom-Made Database, Biomarker Assignment, or Mathematical Classifiers Does Not Differentiate Shigella spp. and Escherichia coli
Source: Microorganisms. 2022 Feb 14;10(2):435. doi: 10.3390/microorganisms10020435 (PMC8878589; doi:10.3390/microorganisms10020435)
Supplement: Supplementary file 1 [file microorganisms-10-00435-s001.zip › Supplementary Material.pdf]

Supplementary Figure S1. Decision diagrams of assigned biomarkers
a.Biomarkers at genus level. b. Biomarkers at pathotype level. c. Biomarkers at group level

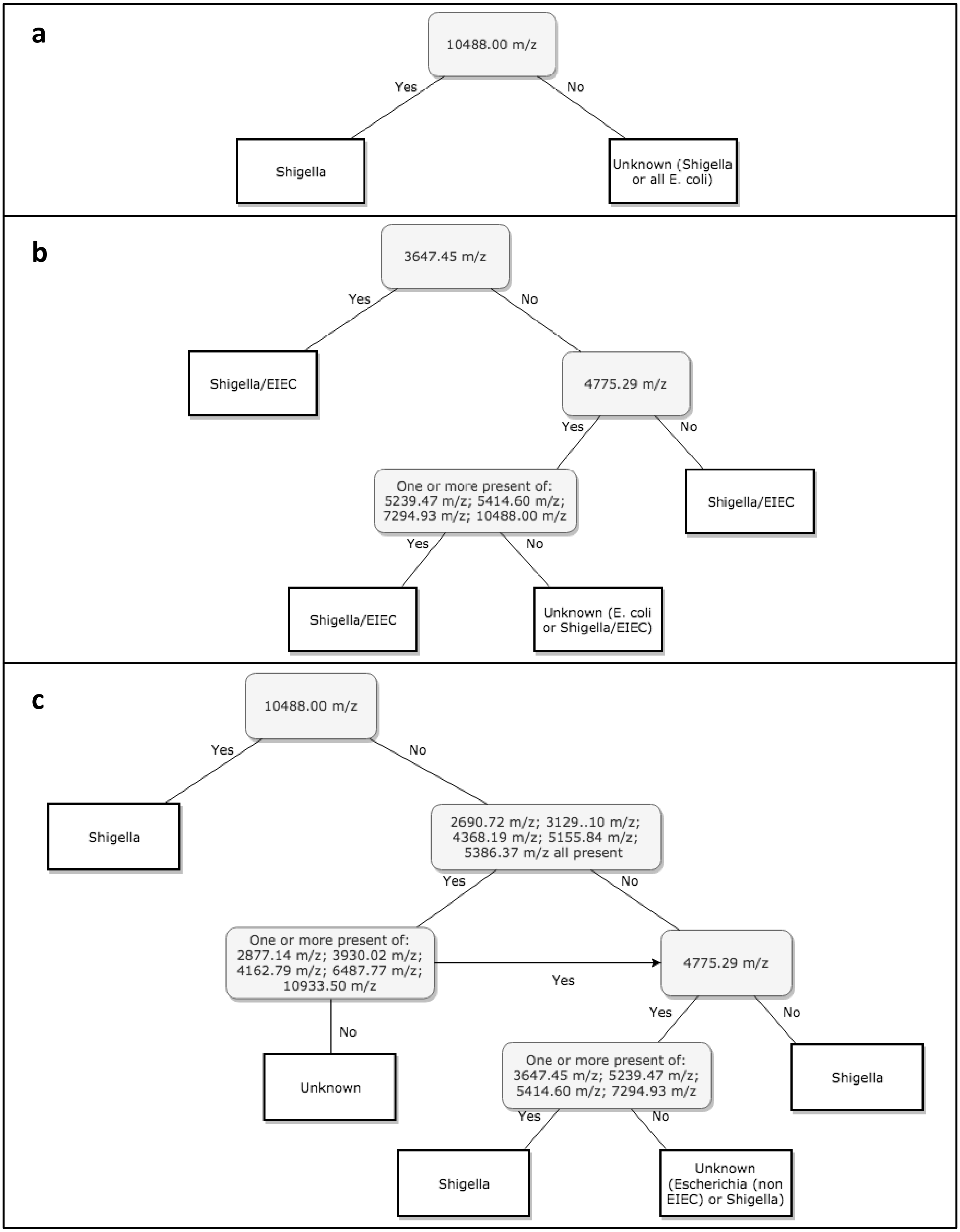

Supplementary Table S1. Comparison of performance of a custom-made database and classifiers based on all 288 isolates or based on an evenly distribution of 17 isolates for each species (6*17)

	**Correct identification with MALDI-TOF, direct smear:**				**Correct identification with MALDI-TOF, ethanol-formic acid extraction:**			
	**Custom-made database, training set 288 isolates n (%)**	**Custom-made database, training set 6*17 isolates n (%)**	**Classifier models, training set 288 isolates n (%)**	**Classifier models, training set 6*17 isolates n (%)**	**Custom-made database, training set 288 isolates n (%)**	**Custom-made database, training set 6*17 isolates n (%)**	**Classifier models, training set 288 isolates n (%)**	**Classifier models, training set 6*17 isolates n (%)**
**Genus**								
***Shigella* (n =217)**	205 (94)	196 (90)	209 (96)	157 (72)	207 (95)	201 (93)	217 (100)	191 (88)
***E. coli* (n = 52)**	26 (50)	28 (54)	11 (21)	11 (21)	35 (67)	27 (52)	4 (8)	9 (17)
**Unassigned**	1 (0.4)	1 (0.4)	0 (0)	0 (0)	0 (0)	2 (1)	0 (0)	0 (0)
**Pathotype**								
***Shigella*/EIEC (n = 248)**	233 (94)	222 (90)	145 (58)	157 (63)	245 (99)	241 (97)	147 (59)	242 (96)
**Other *E. coli* (n =21)**	6 (29)	10 (48)	14 (67)	1 (5)	11 (52)	9 (43)	6 (29)	1 (5)
**Unassigned**	1 (0.4)	1 (0.4)	0 (0)	0 (0)	0 (0)	2 (1)	0 (0)	0 (0)
**Group**								
***Shigella* (n = 217)**	205 (94)	196 (90)	131 (60)	142 (65)	207 (95)	201 (93)	134 (62)	208 (96)
**EIEC (n = 31)**	9 (29)	5 (16)	2 (6)	1 (3)	19 (61)	11 (35)	0 (0)	1 (3)
**Other *E. coli* (n =21)**	6 (29)	10 (48)	13 (62)	1 (5)	11 (52)	9 (43)	7 (33)	1 (5)
**Unassigned**	1 (0.4)	1 (0.4	0 (0)	0 (0)	0 (0)	2 (1)	0 (0)	0 (0)
**Species**								
***S. dysenteriae* (n =11)**	5 (45)	5 (45)	0 (0)	0 (0)	7 (64)	7 (64)	0 (0)	1 (9)
***S. flexneri* (n =77)**	70 (91)	59 (77)	6 (8)	0 (0)	73 (95)	64 (83)	3 (4)	0 (0)
***S. boydii* (n =14)**	1 (7)	4 (29)	0 (0)	0 (0)	0 (0)	3 (21)	0 (0)	0 (0)
S. sonnei (n = 115)	110 (96)	105 (91)	92 (80)	60 (52)	112 (97)	112 (97)	101 (88)	113 (98)
**EIEC (n = 31)**	9 (29)	5 (16)	1 (3)	2 (6)	19 (61)	11 (35)	3 (10)	4 (13)
**Other *E. coli (n =21)***	6 (29)	10 (48)	12 (57)	2 (10)	11 (52)	9 (43)	4 (19)	4 (19)
**Unassigned**	1 (0.4)	1 (0.4)	0 (0)	0 (0)	0 (0)	2 (1)	0 (0)	0 (0)

Percentage correct identification of total isolates (n =269) is displayed.
